# Phylogeny rather than ecology or lifestyle biases the construction of *Escherichia coli*–*Shigella* genetic exchange communities

**DOI:** 10.1098/rsob.120112

**Published:** 2012-09

**Authors:** Elizabeth Skippington, Mark A. Ragan

**Affiliations:** Institute for Molecular Bioscience and Australian Research Council Centre of Excellence in Bioinformatics, The University of Queensland, Brisbane, Queensland 4072, Australia

**Keywords:** lateral genetic transfer, horizontal genetic transfer, genetic exchange communities

## Abstract

Genetic material can be transmitted not only *vertically* from parent to offspring, but also *laterally* (*horizontally*) from one bacterial lineage to another. Lateral genetic transfer is non-uniform; biases in its nature or frequency construct communities of genetic exchange. These biases have been proposed to arise from phylogenetic relatedness, shared ecology and/or common lifestyle. Here, we test these hypotheses using a graph-based abstraction of inferred genetic-exchange relationships among 27 *Escherichia coli* and *Shigella* genomes. We show that although barriers to inter-phylogenetic group lateral transfer are low, *E. coli* and *Shigella* are more likely to have exchanged genetic material with close relatives. We find little evidence of bias arising from shared environment or lifestyle. More than one-third of donor–recipient pairs in our analysis show some level of fragmentary gene transfer. Thus, within the *E. coli*–*Shigella* clade, intact genes and gene fragments have been disseminated non-uniformly and at appreciable frequency, constructing communities that transgress environmental and lifestyle boundaries.

## 2. Introduction

Lateral genetic transfer (LGT; also known as horizontal genetic transfer, HGT) has significantly reshaped the genetic repertoires of many prokaryotic genomes [1–4]. In particular, genetic determinants of pathogenicity and other adaptive traits can spread rapidly via LGT. Genetic exchange communities (GECs) are sets of genomes that mutually exchange genetic information via LGT [5].

Much remains to be understood about the structure and interrelationships of GECs, and about the frequency and nature of LGT within them. Genetic-exchange relationships can be abstracted as a graph in which nodes represent entities that carry genetic material (e.g. bacterial genomes and vectors), and edges represent pairwise transfer (whether vertical, i.e. from parents to offspring, or lateral) of genetic material between them. We defined GECs as densely connected regions within such a network, where the edges reflect LGT [5]. Examining the topological properties of such subgraphs should allow us to formulate and test the hypotheses regarding the nature and dynamics of genetic exchange that constructs such communities.

GECs do not exist *a priori* in nature, but rather are constructed through contingencies, biases and barriers that shape the interplay between donor and recipient lineages in dynamic environments [5]. Several hypotheses regarding transfer bias have been proposed, notably that successful LGT is more frequent *within* than *between* taxonomic groups [6–11], or where donor and recipient share a common environment or ecological niche [9,12]. At least for closely related donors and recipients, genes are transferred more frequently between pathogens than between non-pathogenic species [13].

The *Escherichia coli*–*Shigella* clade is an attractive testbed for hypotheses of LGT bias, as complete genomes of environmentally and physiologically diverse strains, including commensals as well as intra- and extra-intestinal pathogens, have been sequenced and annotated. Genomes within the clade differ remarkably in gene content, and LGT appears to have been frequent [4,14–16]. An analysis of 5282 sets of orthologous protein-coding genes from 27 strains of *E. coli*–*Shigella* [17] revealed evidence for LGT in 2655 (50.3%) sets, of which 678 (12.8%) contained one or more internal recombination breakpoints indicative of fragmentary (within-gene) LGT.

Using these data as a starting point, we have now inferred directed pathways of LGT among these 27 genomes, and abstracted them as a graph. Applying concepts from graph theory, we delineate GECs and examine the pathways, biases and frequencies of transfer that construct them. This allows us to consider whether our operational definition of a GEC [5] is appropriate, and to test hypotheses that gene transfer occurs preferentially within phyletic groups, within a common environment and/or within a lifestyle (e.g. among pathogens). Finally, we investigate the units of transfer that circulate within these GECs by examining the presence or absence of recombination breakpoints within gene sets that are topologically discordant with the *E. coli*–*Shigella* reference phylogeny.

## 3. Results and discussion

### 3.1. Inference of phylogenetic discordance and recombination breakpoints among gene sets

These 27 genomes sample the phylogenetic and environmental diversity within *E. coli*–*Shigella* as available when our study was initiated. Twenty genomes from the major *E. coli* phylogenetic groups A, B1, B2, D and E, and seven from the closely related *Shigella* (table 1), reflect a breadth of environments, adaptive challenges and lifestyles, including commensal, entero- and extra-intestinal pathogenic lineages.

**Table 1.** The 27 *Escherichia coli* and *Shigella* genomes used in this study. NCBI, National Center for Biotechnology Information.

genome	NCBI identifier	clinical condition and/or pathotype^a^	reference(s)
*E. coli* SE11	NC_011415	commensal	[18]
*E. coli* IAI1	NC_011741	commensal	[14]
*E. coli* 55989	NC_011748	diarrhoea (EAEC)	[14]
*E. coli* E24377A	NC_009801	diarrhoea (ETEC)	[19]
*Shigella boydii* Sb227	NC_007613	shigellosis	[20]
*Shigella boydii* CDC 3083 94	NC_010658	shigellosis	[21]
*Shigella sonnei* Ss046	NC_007384	shigellosis	[20]
*Shigella flexneri* 2a 2457T	NC_004741	shigellosis	[22]
*Shigella flexneri* 2a	NC_004337	shigellosis	[23]
*Shigella flexneri* 5 8401	NC_008258	shigellosis	[24]
*E. coli* C ATCC 8739	NC_010468	commensal	[25]
*E. coli* HS	NC_009800	commensal	[19]
*E. coli* K12 substr MG1655	NC_000913	commensal	[26]
*E. coli* K12 substr W3110	AC_000091	commensal	[27]
*E. coli* 0157 : H7 EDL933	NC_002655	diarrhoea (EHEC)	[28]
*E. coli* O157 : H7	NC_002695	diarrhoea (EHEC)	[29]
*Shigella dysenteriae*	NC_007606	shigellosis	[20]
*E. coli* UMN026	NC_011751	cystitis (ExPEC)	[14]
*E. coli* APEC O1	NC_008563	colisepticaemia (ExPEC, APEC)	[30]
*E. coli* S88	NC_011742	newborn meningitis (ExPEC)	[14]
*E. coli* UTI89	NC_007946	cystitis (ExPEC, UPEC)	[31]
*E. coli* ED1a	NC_011745	healthy subject	[14]
*E. coli* 536	NC_008253	pyelonephritis (ExPEC, UPEC)	[32,33]
*E. coli* CFT073	NC_004431	pyelonephritis (ExPEC, UPEC)	[34]
*E. coli* 0127 H6 E2348 69	NC_011601	diarrhoea (EPEC)	[35]
*E. coli* IAI39	NC_011750	pyelonephritis (ExPEC)	[14]
*E. coli* SMS 3 5	NC_010498	environmental strain	[36]

^a^EAEC, enteroaggregative *E. coli*; ETEC, enterotoxigenic *E. coli*; EHEC, enterohemorrhagic *E. coli*; ExPEC, extraintestinal pathogenic *E. coli*; APEC, avian pathogenic *E. coli*; UPEC, uropathogenic *E. coli*; EPEC, enteropathogenic *E. coli*.

**Figure 1.**
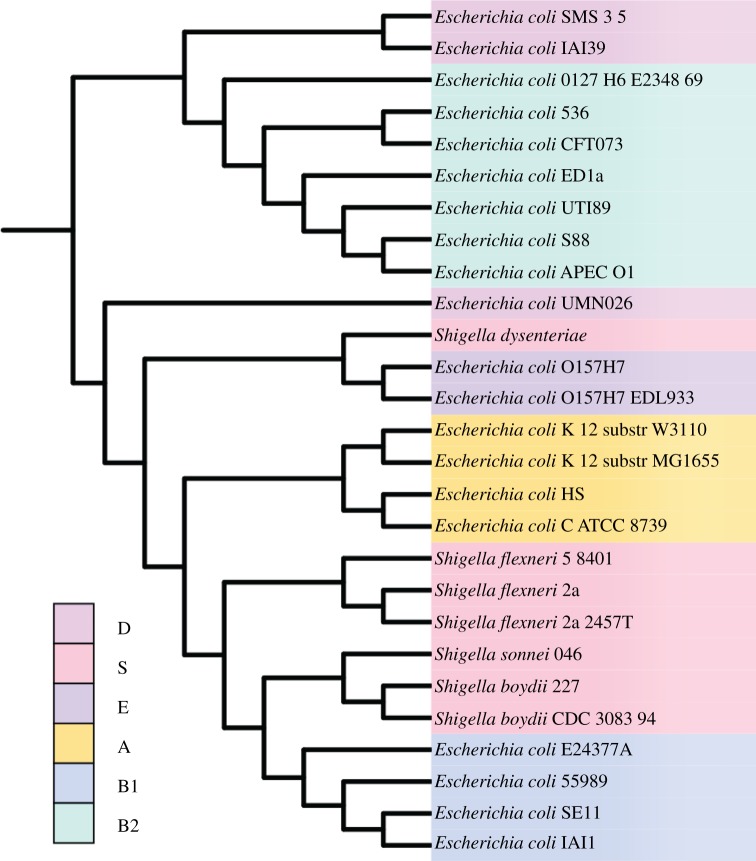
The *E. coli*–*Shigella* reference supertree, constructed using matrix representation with parsimony [38] based on well-supported bipartitions in 5282 Bayesian protein trees. Colours indicate membership in recognized *E. coli* phylogenetic groups.

Using whole-genome alignment, we delineated 5282 sets of proteins with at most one member per genome (i.e. putative orthologues) and size *n* ≥ 4, and for each we inferred a Bayesian phylogenetic tree [37]. Aggregating all well-supported bipartitions (posterior probability, PP ≥ 0.95) using matrix representation with parsimony (MRP) [38] yielded a robust reference topology for *E. coli*–*Shigella* (figure 1). This MRP tree is remarkably concordant with the *E. coli*–*Shigella* phylogeny reported by Touchon *et al.* [14], which they inferred by maximum likelihood from 1878 concatenated *E. coli*–*Shigella* core gene sequences ([17], fig. 4). Of the 52 bipartitions in our MRP tree, 49 appear in the Touchon *et al.* tree. Both trees support the monophyly of all classical groups described by multi-locus enzyme electrophoresis [39] except for phylogenetic group D, which both we and Touchon *et al.* [14] recover as polyphyletic.

Individual protein trees with at least one well-supported (PP ≥ 0.95) bipartition topologically incongruent with this MRP reference tree provide evidence of intra-clade LGT. Of the 5282 protein trees, we found 2440 (46.2%) to have at least one incongruent bipartition, vis-à-vis the MRP reference, at PP ≥ 0.95. From an analysis of 144 genomes representing 15 prokaryotic phyla, Chan *et al.* [40] reported strong evidence for LGT among 342 (23.4%) of 1462 orthologous gene sets. The higher proportion of putative LGT we detect within *E. coli*–*Shigella* may reflect the relatively greater frequency with which exogenous DNA can be integrated via homologous recombination [41].

### 3.2. Pathways of lateral genetic transfer among *Escherichia coli* and *Shigella* strains inferred from phylogenetic discordance

Discordance between a gene or protein (*query*) tree and a reference tree can be reconciled by carrying out one or more subtree prune-and-regraft operations on the latter. Beiko *et al.* [42] refer to these operations individually as *edits*, a connected series of which traces an *edit path* that constitutes a hypothesis of LGT between two genomes. Using Efficient Evaluation of Edit Paths (EEEP) [43], we inferred the edit paths that most parsimoniously reconcile incongruence between well-supported (PP ≥ 0.95) bipartition(s) of individual protein trees and our MRP reference. Such comparisons sometimes yield multiple possible reconciliation paths; following Beiko *et al*. [42], we refer to an edit as *obligate* if it is implied by every path in the set of most-parsimonious reconciliation paths, and *possible* if it is implied by some, but not necessarily all, such paths. Obligate edits can thus be viewed as high-confidence hypotheses of lateral transfer. In contrast with Beiko *et al.* [42], here we consider only those obligate edits for which the direction of transfer can be inferred. Interpreting paths that involve non-obligate edits is much more difficult, and remains a key challenge in studying LGT [43]. Topological reconciliation on a tree is NP-hard; so especially when the pattern of incongruence is complex, it may not be possible to compute a minimal edit path. Nonetheless, we found most parsimonious edit paths for 2389 (97.9%) of the 2440 of the incongruent protein trees, 472 (19.8%) of which gave rise to at least one directional obligate edit.

### 3.3. A directed network of obligate lateral genetic transfer among strains of *Escherichia coli* and *Shigella*

The set of unique connections implied by the obligate edits can be abstracted as a network, within which GECs can then be delineated [5]. The LGT network we develop here is non-standard in certain ways. Because we infer edits by reference to a (temporal) phylogenetic tree, a node in our abstracted network may represent an extant genome (i.e. a branch-tip), or one inferred as ancestral (an internal branch-point including its immediately subtending edge). Much genetic material has of course been inherited vertically, hence transmitted within (not across) a cellular lineage; but as here we are concerned only with LGT, in abstracting the network, we disregard connections that appear as edges in the MRP supertree. We likewise disregard all connections that describe genetic material as flowing backwards in time. Thus, each edge in our abstracted network connects partners in an obligate edit that resolves incongruence for at least one protein (query) set. This graphical abstraction necessarily flattens out the temporal diversity of genetic-exchange relationships within the clade [5]. The resulting edges may be unidirectional (one partner has always been the donor) or bidirectional (both exchange partners have donated to, and accepted from, the other), and can be further labelled by frequency of transfer (see §3.5). Sister termini (genomes) in the MRP tree are never directly connected in our network, as it is not possible to infer LGT between sister termini using a topological approach.

We summarized all obligate edits as a *directed obligate LGT network*, or DOLN. The DOLN we infer for these 27 genomes consists of 52 vertices (27 extant and 25 ancestral) connected by 462 edges. It is a graphical representation of the high-confidence LGT network within the *E. coli–Shigella* clade. Evidence for each individual edge is provided by (one or more) protein sets; paths through the DOLN thus inform more broadly about directed LGT within the clade over time, without necessarily reflecting the history of any gene set individually. Extracting the obligate edits from the set of all possible edit paths represented a substantial filtering: the 2389 resolved incongruent protein trees gave rise to 1925 unique possible edits, of which 462 (24%) are obligate. Although this may introduce bias, no principled basis has been described for interpreting transfer histories from sets of non-obligate edits [43].

### 3.4. Topological properties of the *Escherichia coli–Shigella* obligate lateral genetic transfer network

Strains of *E. coli* and *Shigella* have accepted large amounts of genetic material from external lineages [4,14,15] and are thus members of one or more GECs more inclusive than this clade itself. Our *E. coli–Shigella* DOLN comprises 46 recipient and 46 donor genomes (extant and ancestral), with 44 inferred as both; a further four genomes (*E. coli* K-12 W3110, K-12 MG1655 and O157 : H7, and *S. flexneri* 2457T) were not implicated by any obligate edit, and thus fall outside the connected component of the DOLN. *E. coli* O157 : H7, for example, is known to have acquired a large amount of genetic material via LGT [44], but was not implicated as a transfer partner by any obligate edit. Note that (i) we did not consider genes carried on plasmids; (ii) high sequence similarity (for example, between *E. coli* O157 : H7 and *E. coli* O157 : H7 EDL933) makes it difficult to resolve, hence distinguish, branching structure within trees; and (iii) using a phylogenetic approach, LGT cannot be inferred for protein sets of size *n* < 4, nor for adjacent terminal genomes. Our DOLN corresponds to a single subgraph that, although densely connected, nonetheless allows us to recognize even more densely connected regions, and thereby to survey features of the intra-specific LGT events that construct GECs.

**Figure 2.**
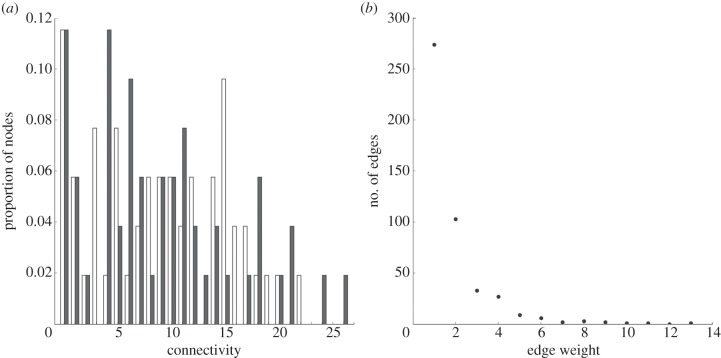
Properties of the directed obligate LGT network. Distribution of (*a*) connectivity and (*b*) edge labels. Filled bars denote out-degree; unfilled bars, in-degree.

We have recommended that a GEC be defined as *a set of entities, each of which has over time both donated genetic material to, and received genetic material from, every other entity in that GEC, via a path of lateral transfer* [5]. Does our *E. coli–Shigella* DOLN (figure 2) meet this stringent criterion? Recall that edges cannot be realized between sister termini, and that genetic material cannot flow backwards in time. Of 2072 potential edges, we observe only 462 (22.3%); however, 2072 (78.1%) of the 2704 possible node pairs are connected by a path of length greater than or equal to 1; that is, our *E. coli*–*Shigella* DOLN is densely connected, but, by our proposed criterion (above), falls short of qualifying as a GEC. Of the missing edges, many would connect closely related genomes (*E. coli* K-12 W3110 with K-12 MG1655; *E. coli* EDL933 with O157 : H7; strains within *S. flexneri*) among which LGT can often not be inferred owing to high sequence similarity. Including all *possible* (not only obligate) edits greatly increases the density of connection: 1925 (93%) of 2072 possible node pairs are connected by a path of length 1, and all possible node pairs (2072/2072, 100%) by a path of length greater than or equal to 1. Our inference may have missed other paths owing to incomplete or uneven sampling of genomes. Although the inferred connectivity of the DOLN falls short of our proposed criterion, we suspect, for the above reasons, that the *E. coli*–*Shigella* clade is in fact a GEC. This will be testable as more genomes in this clade are sequenced.

**Figure 3.**
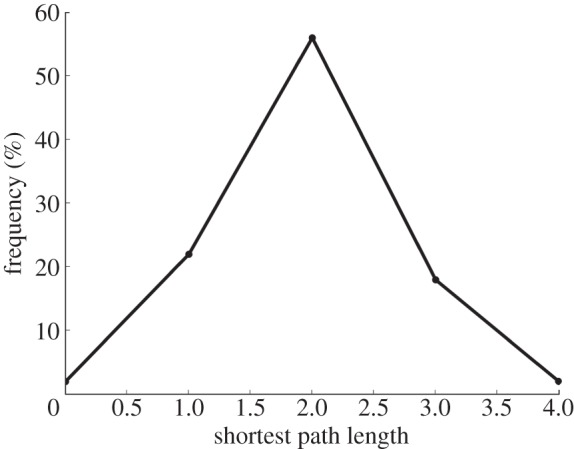
Distribution of shortest paths for the directed obligate LGT network. Zero-length paths represent self-connections (e.g. genome A to itself).

The shortest paths between genome pairs in our DOLN (figure 3) range from zero to four steps in length, with most (1610/2072, 77.7%) pairs connected by a path of length more than or equal to 2. Although somewhat inflated because LGT cannot be inferred between sister termini, this proportion nonetheless indicates a breadth of genetic connectivity across the clade, expanding the possibility for DNA to flow into groups or communities not accessible in a single step from the donor genome.

Unlike many other biological networks [45], the *E. coli*–*Shigella* DOLN is not scale-free and does not contain *hubs* (nodes that are far more highly connected than most). The number of recipient genomes per donor (out-degree) ranges from 1 to 26, and the number of donating genomes per acceptor (in-degree) from 1 to 22 (figure 2). In-degree and out-degree are correlated (*r* = 0.74, *p* < 0.001); thus any given *E. coli* or *Shigella* genome has both donated genetic material to, and received genetic material from, a comparable number of other genomes.

**Figure 4.**
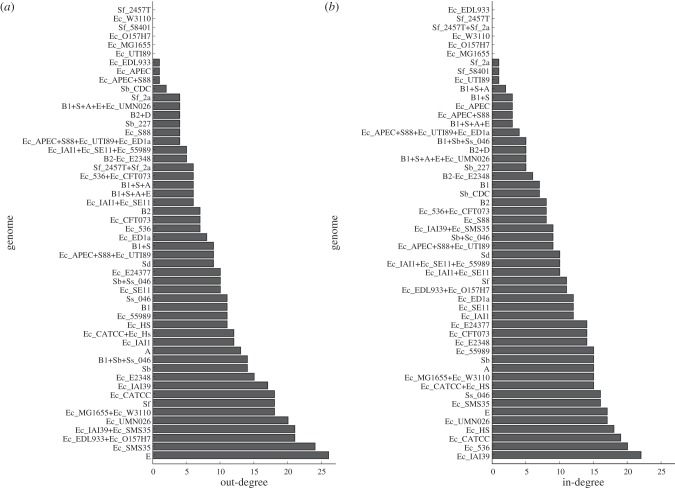
Distribution of connectivity by branch for the directed obligate LGT network. (*a*) Donors, (*b*) recipients.

**Figure 5.**
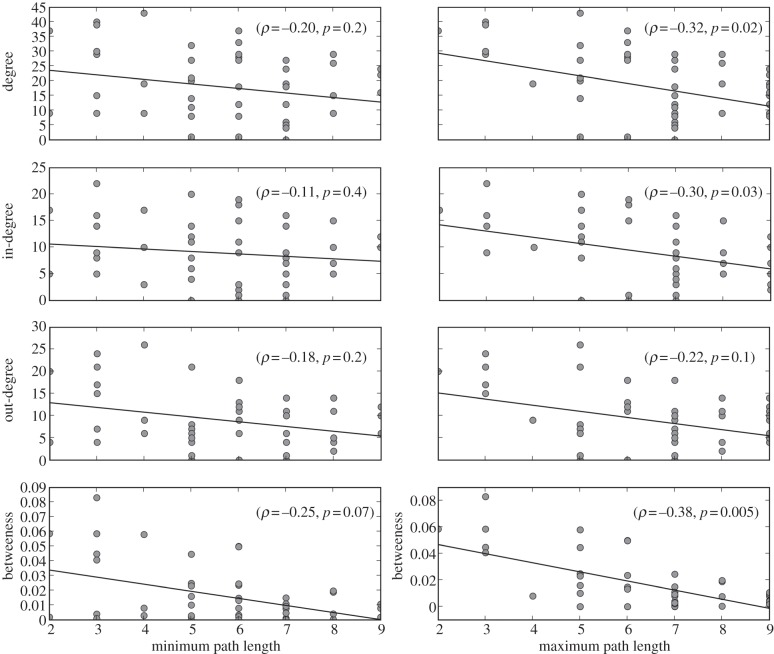
Degree and betweenness of nodes (genomes) of the directed obligate LGT network (DOLN) as a function of the lengths of the corresponding shortest and longest root-to-leaf paths in the MRP tree. Because we inferred LGT by reference to a (temporal) phylogenetic tree, a node in the DOLN may represent an extant genome, or one inferred as ancestral in the MRP tree. Because there exists a path from every leaf in the MRP tree to the root, ancestral genomes fall on one or more of these paths. Scatterplots show weak to moderate downward trends. Black lines are best-fit first-order polynomials; rho (*ρ*) is the Spearman rank correlation coefficient.

Among these extant genomes, environmental strain *E. coli* SMS-3–5 has donated genetic material to the largest number of other genomes, while the extra-intestinal pathogen *E. coli* IAI39 has accepted genetic material from the largest number (figure 4). As relatively few nodes separate each of these from the root of the MRP reference tree, it could be that their apparently heightened involvement in LGT is an artefact of sampling bias. To assess this possibility, for each genome in the MRP tree, we plotted the length of the shortest and of the longest root-to-leaf path on which it falls (as surrogates for sampling density in that portion of the MRP tree) against measures of connectedness (figure 5). Each plot shows a weak to moderate downward trend in the data. Specifically, Spearman's rank correlation coefficients for the comparison between path length and each of the connectivity measures range from −0.38 (betweenness versus maximum path length) to −0.11 (in-degree versus minimum path length). Thus, we find little evidence to suggest that relative phyletic coverage has affected our recovery of edit paths.

**Figure 6.**
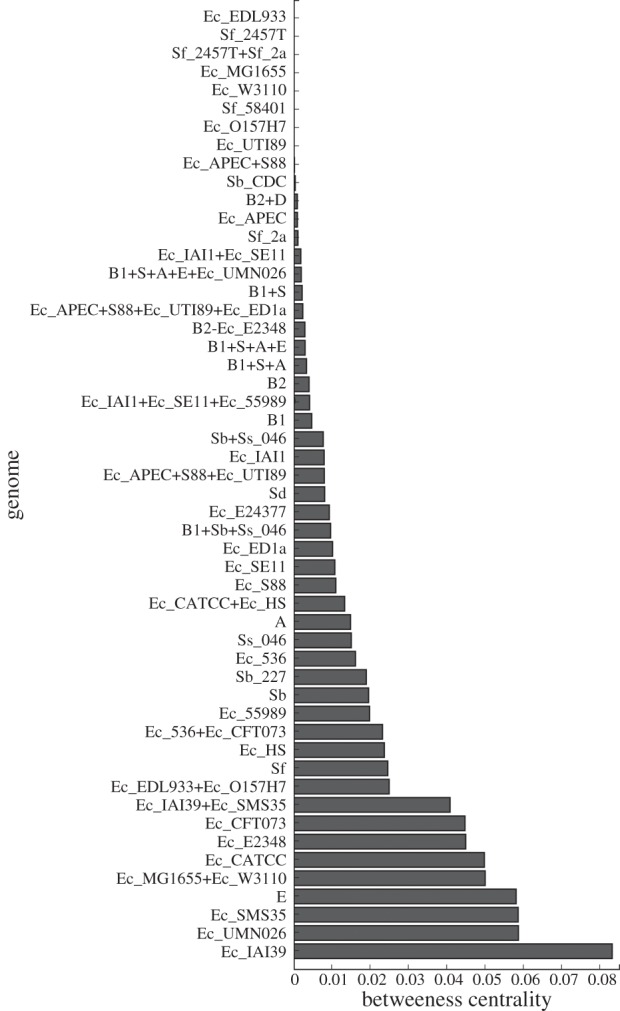
Distribution of betweenness centrality for nodes of the directed obligate LGT network (DOLN).

*Betweenness* is a measure of node centrality defined [46] as the frequency at which a given node lies on the shortest path between any pair of nodes in a network. High-betweenness nodes are important because they intermediate between genomes, other genetic entities and/or communities that do not exchange genes directly. Figure 6 shows the distribution of betweenness centrality for nodes of our *E. coli*–*Shigella* DOLN. The three genomes with highest node betweenness are the environmental strain *E. coli* SMS-3–5, and the extra-intestinal pathogens *E. coli* IAI39 and UMN026. Although this measure shows a moderate degree of potential sampling bias (figure 5), high-betweenness nodes clearly are present in our DOLN. We next investigate their potential role in constructing the *E. coli*–*Shigella* GEC, focusing on frequencies and pathways of transfer within and between phyletic groups, lifestyles and habitats.

### 3.5. Differential transfer frequencies reveal patterns of lateral genetic transfer that reinforce traditional phylogenetic groupings but link distinct bacterial lifestyles

Differential frequencies of LGT can be important in constructing GECs [5]. To assess how evenly successful transfers are distributed across the *E. coli*–*Shigella* DOLN, we labelled each edge with the number of incongruent protein sets whose resolution requires that edge as an obligate edit. The value of an edge label corresponds to the number of inferred transfer events between the donor and recipient lineages connected by that edge in our DOLN. The sum of all edge label values (i.e. the total number of inferred obligate transfers involving protein-coding genes) is 858. Because we infer shortest edit paths, these edge labels reflect the *minimum* number of such transfers. The number of obligate transfers per edge ranges from 1 to 13 (figure 2); more than half of the edges (274/462, 59%) reflect a single obligate transfer, while only 25 (5%) represent five or more.

**Figure 7.**
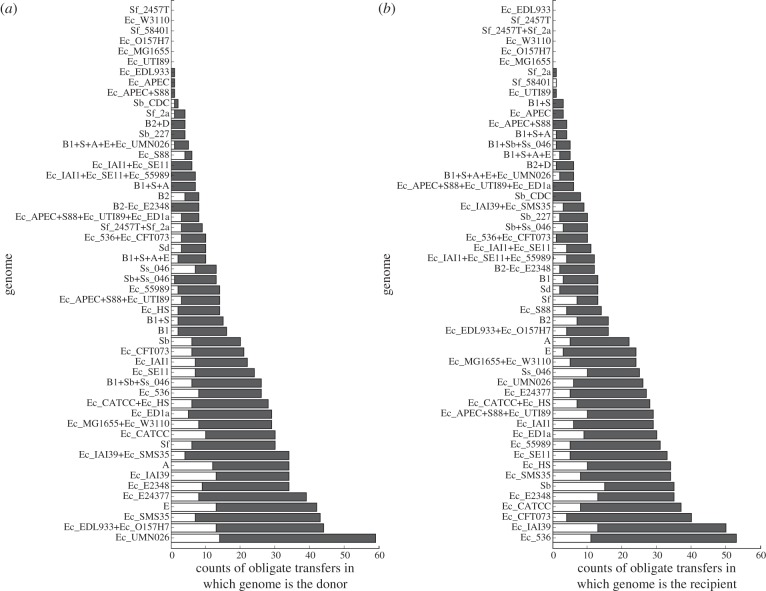
Distribution of obligate transfers by (*a*) donor and (*b*) recipient genomes. Presence (ORB+) or absence (ORB−) of within-gene recombination breakpoints in discordant protein sets that give rise to obligate transfers are represented by white and grey bars, respectively.

Summing the label values of all outgoing and incoming edges gives the number of obligate transfers that implicate that node as a donor or as a recipient, respectively (figure 7). *E. coli* UMN026 is the most frequent donor genome among the obligate transfers, while *E. coli* 536 is the most frequent recipient*.* Both strains are extra-intestinal pathogens.

**Figure 8.**
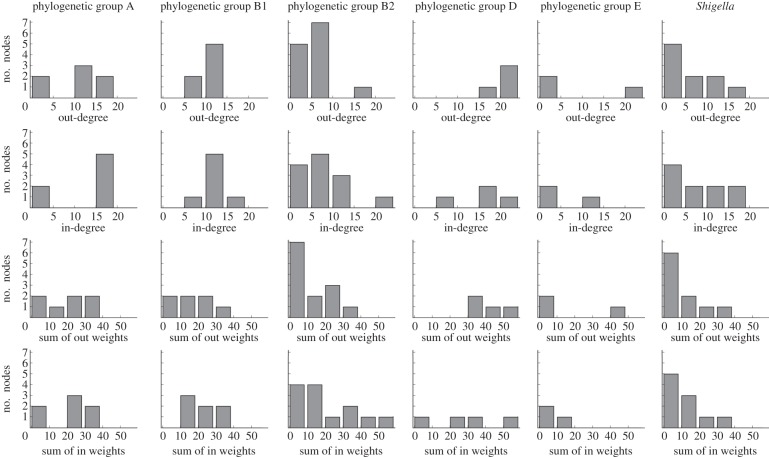
Connectivity and edge label value distribution of the directed obligate LGT network by *E. coli* phylogenetic groups.

Edges with label values greater than or equal to 5 are listed in table 2. The donor–recipient pairs with greatest total edge label are *E. coli* strains E24377A and IAI1, *E. coli* strains ED1a and CFT073, and *E. coli* strains E24377A and SE11. Each of these connections crosses recognized phylogenetic groups, and links a commensal with a pathogenic strain: barriers to transfer across groups and lifestyles can be low. On the other hand, 11 of 16 edges with label greater than or equal to 6 (69%) reflect intra-group transfer, consistent with the ready integration of incoming DNA *via* homologous recombination. Differing edge weight and connectivity distributions were observed across the *E. coli* phylogenetic groups and *Shigella* (figure 8). We next investigate the number and diversity of exchange partners, and frequencies of transfer, within and between phylogenetic groups.

**Table 2.** Connections that have edge labels in the range five to thirteen in the directed obligate LGT network (DOLN).

donor genome	donor phylogroup	donor pathotype	recipient genome	recipient phylogroup	recipient pathotype	minimum number of genes transferred (edge weight)
*E. coli* E24377A	B1	EPEC	*E. coli* IAI1	B1	commensal	13
*E. coli* ED1a	B2	healthy subject	*E. coli* CFT073	B2	ExPEC	11
*E. coli* E24377A	B1	EPEC	*E. coli* SE11	B1	commensal	10
*E. coli* UMN026	D	ExPEC	*E. coli* E2348	B2	EPEC	9
*E. coli* SMS35	D	environmental	*E. coli* 536	B2	ExPEC	9
*E. coli* CFT073	B2	ExPEC	*E. coli* APEC+*E. coli* S88+*E. coli* UTI89	B2 (ancestral)	—	8
*E. coli* ED1a	B2	healthy subject	*E. coli* 536	B2	ExPEC	8
*E. coli* UMN026	D	ExPEC	*E. coli* SMS35	D	environmental	8
*E. coli* UMN026	D	ExPEC	*E. coli* IAI39	D	ExPEC	7
*E. coli* C ATCC+*E. coli* HS	A (ancestral)	—	*E. coli* 55989	B1	EPEC	7
*E. coli* 536	B2	ExPEC	*E. coli* APEC+*E. coli* S88+*E. coli* UTI89	B2 (ancestral)	—	6
*E. coli* E2348	B2	EPEC	*E. coli* 536	B2	ExPEC	6
*E. coli* E2348	B2	EPEC	*E. coli* CFT073	B2	ExPEC	6
*E. coli* EDL933+*E. coli* O157 : H7	E (ancestral)	—	*S. boydii*	*S. boydii* (ancestral)	—	6
*E. coli* IAI1	B1	commensal	*E. coli* E24377A	B1	EPEC	6
*E. coli* SE11	B1	commensal	*E. coli* HS	A	commensal	6
*E. coli* UMN026	D	ExPEC	*E. coli* MG1655+*E. coli* W3110	A (ancestral)	—	5
*E. coli* EDL933+*E. coli* O157 : H7	E (ancestral)	—	*E. coli* C ATCC	A	commensal	5
*E. coli* UMN026	D	ExPEC	*E. coli* 536	B2	ExPEC	5
A	A (ancestral)	—	*E. coli* IAI39	D	ExPEC	5
A	A (ancestral)	—	*S. boydii*	*S. boydii* (ancestral)	—	5
*E. coli* C ATCC+*E. coli* HS	A (ancestral)	—	*S. boydii*	*S. boydii* (ancestral)	—	5
*E. coli* 536	B2	ExPEC	*E. coli* ED1a	B2	healthy subject	5
*E. coli* CFT073	B2	ExPEC	*E. coli* ED1a	B2	healthy subject	5
*E. coli* SE11	B1	commensal	A	A (ancestral)	—	5

### 3.6. Biased gene transfer *within* phylogenetic groups

Genomes from each of the four major *E. coli* phylogenetic groups (A, B1, B2, D) and from accessory group E [39,47,48] are represented in our DOLN. Our MRP tree reconstructs each of these groups as monophyletic except group D, which, in agreement with Touchon *et al*. [14], we recover as polyphyletic. Strains in these groups differ in ecological niche, lifestyle and presence of virulence factors: most commensal strains belong to groups A and B1, extra-intestinal pathogens to groups B2 and D, and intestinal pathogens to groups B1 and D [49], while strains of groups B2 and D frequently harbour virulence factors not present in groups A and B1 [50]. We examined the paths of genetic flow within and between these groups.

Ancestral genomes were assigned to a phylogenetic group if, in our MRP tree, all their extant descendants belong to the same group. Nine ancestral genomes have extant descendants in more than one phylogenetic group; we refer to these ancestral genomes as *unclassified*, and do not consider them in detail. The DOLN reveals extensive LGT between *E. coli* phylogenetic groups, but fewer connections within groups. Among the 462 edges in our DOLN network, 45 (10%) connect a donor and recipient within the same group, while 312 (68%) are inter-group (the remaining 105 edges involve at least one unclassified partner). Although not bias-free (see §3.3), these results clearly indicate that within the *E. coli*–*Shigella* GEC, barriers to inter-group LGT are low. They probably, however, underestimate the true extent of LGT within groups, as our approach is blind to transfer between terminal sister lineages. Normalizing the counts of intra-group and inter-group edges by dividing each total count by the number of possible edges within the group, we find that the frequency of intra-group edges (45/201 = 0.21) is almost identical to that of inter-group edges (312/1530 = 0.20).

Compared with the inter-group edges, the intra-group edges have larger label values (*p* < 0.001 by Wilcoxon rank sum test; 44 intra- and 312 inter-group edges), suggesting a higher number of individual transfer events between donor and recipient lineages that belong to the same phylogenetic group. At broader taxonomic scales, LGT is more frequent within than between taxonomic groups [6,10,11,13]; our results suggest that this is also the case (at least for *E. coli*–*Shigella*) at the sub-specific level. Among intra-group edges, those linking phylogenetic group A with B1, and B2 with D, are most frequent (table 3). As most commensal strains are in groups A and B1 [50], and most extra-intestinal pathogens in groups B2 and D [49], this raises the possibility of preferential transfer among strains that share a similar lifestyle.

**Table 3.** Frequency and count label of obligate lateral edges by intra-phylogenetic group and inter-phylogenetic group subsets. Frequency is calculated by dividing the count of obligate edges by the number of possible within-group connections. The median and ranges for edge labels are shown in parentheses.

	A	B1	B2	D	E	*Shigella*
A	0/16 = 0					
B1	51/98 = 0.52 (2, 1–7)	4/12 = 0.33 (8, 2–13)				
B2	24/182 = 0.13 (1, 1–4)	7/182 = 0.04 (1, 1–2)	23/76 = 0.30 (4, 1–11)			
D	22/56 = 0.39 (1, 1–5)	22/56 = 0.39 (1, 1–3)	51/102 = 0.50 (2, 1–9)	5/6 = 0.83 (4, 2–8)		
E	5/42 = 0.12 (1, 1–5)	6/42 = 0.14 (1, 1–3)	7/78 = 0.09 (1, 1–4)	4/24 = 0.17 (1.5, 1–2)	none possible	
*Shigella*	33/132 = 0.25 (2, 1–5)	36/162 = 0.22 (1, 1–2)	19/264 = 0.07 (1, 1–3)	16/66 = 0.24 (1, 1–3)	9/44 = 0.20 (2, 1–6)	13/100 = 0.13 (1, 1–4)

### 3.7. Pathways of lateral genetic transfer link *Escherichia coli*–*Shigella* strains with distinct lifestyles and/or living in diverse environments

*Escherichia coli* is a widespread commensal of the human gastrointestinal tract, but the *E. coli*–*Shigella* clade also contains numerous pathogens. Pathogenic *E. coli* strains fall broadly into two groups: extra-intestinal pathogenic *E. coli* (ExPEC) strains [51], which cause urinary tract infections, septis or meningitis in newborns; and intestinal pathogenic *E. coli* (IPEC) strains, which cause enteric diseases. The *Shigella* genomes included here are intestinal pathogens. Our 27 strains include seven commensals, 12 intestinal pathogens and seven extra-intestinal pathogens (environmental strain *E. coli* SMS-3–5 does not fall within any of these groups). Strains in each category face distinct environments and adaptive challenges.

**Figure 9.**
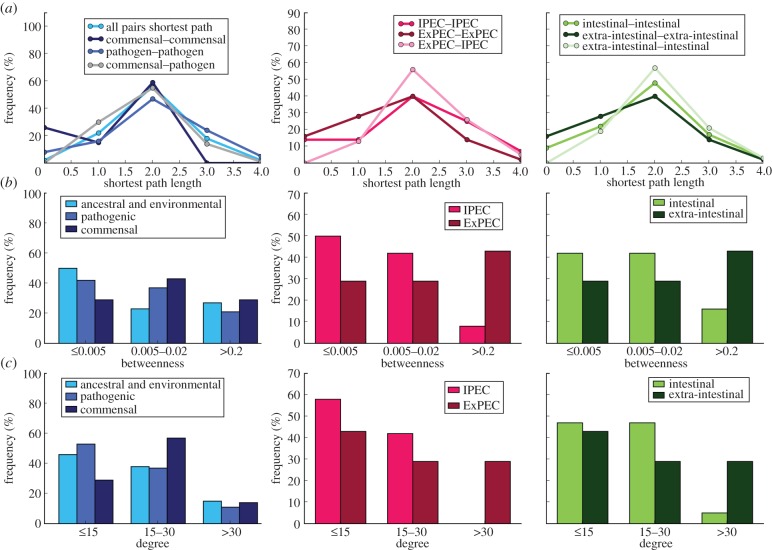
Plots comparing network properties ((*a*) shortest path length, (*b*) betweenness and (*c*) degree) of extant *E. coli* strains by lifestyle and habitat for the directed obligate LGT network (DOLN).

We compared network properties (here, node degree and betweenness) of strains in these categories pairwise to determine whether they distinguish lateral relationships between groups. IPEC and ExPEC strains face distinct environments but share a pathogenic lifestyle. We find no evidence of significant difference between IPEC and ExPEC strains with regard to degree or betweenness (*p* = 0.70 and *p* = 0.48 respectively, by pairwise Wilcoxon rank sum test, Holm-adjusted; figure 9). We similarly compared the commensal strains versus each of these two pathogenic categories. In our DOLN, commensal and IPEC strains exhibit comparable degree (*p* = 1.00) and betweenness centrality (*p* = 0.61, both by pairwise Wilcoxon rank sum test, Holm-adjusted; figure 9). The commensal and ExPEC strains likewise exhibit comparable degree (*p* = 1.00) and betweenness centrality (*p* = 0.61, also by pairwise Wilcoxon rank sum test, Holm-adjusted; figure 9). Thus, these network properties fail to distinguish lateral relationships among these groups of commensal, IPEC and ExPEC strains.

In our DOLN, the node with the highest betweenness centrality represents ExPEC strain *E. coli* IAI39: it has donated genetic material to at least 22 distinct recipients, and accepted from at least 17 unique donors. Most strains we infer as exchange partners of *E. coli* IAI39 correspond to ancestral nodes in the MRP supertree. Among the 11 extant exchange partners of *E. coli* IAI39, five are commensal, four ExPEC and two IPEC. Although these numbers are small, this illustrates that the key pathways of LGT can extend across habitat and lifestyle.

Taking into account that LGT cannot be observed between sister termini and that genetic material cannot flow backwards in time, we considered the counts of intra- and inter-lifestyle edges as a proportion of the number of possible within-group connections (table 4): *commensal*–*pathogenic* (0.18) and *pathogenic*–*pathogenic* (0.12) are slightly more frequent than *commensal*–*commensal* edges (0.11), but we find no evidence that intra- and inter-lifestyle edges represent different numbers of genes (*p* = 0.28 by Wilcoxon rank sum test; 47 inter- and 43 intra-lifestyle edges). Similarly, edge frequencies are appreciable both within (*intestinal* to *intestinal*, 0.13) and between habitats (*intestinal* to *extra-intestinal*, 0.13) (table 5); these frequencies are indistinguishable from each other by label value (*p* = 0.35 by Wilcoxon rank sum test; 34 inter- and 44 intra-habitat edges). For the purpose of this comparison, *extra-intestinal* to *extra-intestinal* edges were not considered to be intra-habitat, as these pathogens associate with different human cell types. Thus, our results indicate that the frequency of LGT among strains of *E. coli–Shigella* is comparable within and across habitats.

**Table 4.** Frequency and count label of obligate lateral edges by lifestyle. Edge frequency is normalized as described in table 3. The median and ranges for edge labels are shown in parentheses.

	commensal	pathogenic
commensal	4/36 = 0.11 (2, 2–6)	
pathogenic	47/266 = 0.18 (2, 1–13)	39/332 = 0.12 (1, 1–9)

**Table 5.** Frequency and count label of obligate lateral edges by habitat. Edge frequency is normalized as described in table 3. The median and ranges for edge labels are shown in parentheses.

	intestinal
intestinal	44/330 = 0.13 (2, 1–13)
extra-intestinal	34/266 = 0.13 (2, 1–11)

### 3.8. Gene fragments are frequently transferred via lateral genetic transfer within genetic exchange communities

Up to this point, we have based our inference of LGT within the *E. coli*–*Shigella* clade on topological conflict between a (protein) query tree and the MRP reference. This approach implicitly takes whole protein-coding genes as the unit of analysis. However, many genes are mosaics of regions with conflicting evolutionary histories [17,40,52,53]. We now distinguish within-gene LGT (transfer of one or more within-gene regions) from whole-gene LGT (transfer of an entire gene or beyond), further classifying the protein sets that yield incongruent phylogenetic trees into these two categories, based on the respective presence or absence of at least one internal recombination breakpoint in the corresponding gene alignment (see §5).

Among the 2440 sets of homologous genes that yield a protein tree incongruent with the reference, we observe strong evidence for at least one recombination breakpoint internal to the open reading frame in 463 (19%); these are instances of within-gene transfer. A further 215 gene sets (not included in the 2440) show clear evidence of one or more internal recombination breakpoints, but the corresponding protein tree is not topologically incongruent; we do not consider these further, as we depend on (the resolution of) incongruence to assign donor and recipient lineages. Of the 472 gene sets that imply at least one obligate edit, 124 (26%) show strong evidence of an internal breakpoint—a higher proportion than for all gene sets that yield incongruent trees (19%).

We classified the edges in our DOLN into two categories: *exclusively observable recombination breakpoint positive* (eORB+), being those for which every gene set that gives rise to that obligate edit exhibits at least one observable recombination breakpoint [40]; and *exclusively observable recombination breakpoint negative* (eORB–), for which no gene set that gives rise to that obligate edit has an observable recombination breakpoint. Of the 462 edges in our DOLN, 290 (63%) are eORB– and 65 (14%) eORB+. Thus, more than half of these obligate edits represent only the transfer of intact genes; many more than represent only the transfer of gene fragments. The remaining 107 (23%) fall into neither category: some, but not all, of the corresponding gene sets exhibit a recombination breakpoint.

Within-gene fragmentary transfer nonetheless contributes significantly to LGT within the *E. coli– Shigella* GEC: 172/462 edges (37%) are implied by at least one topologically discordant ORB+ gene set. Earlier, we examined the distribution of obligate transfers by donor and recipient genomes (figure 7). Most of these genomes, both extant and ancestral, have both donated and accepted at least one gene from an ORB+ gene set (figure 7). These results almost certainly underestimate the contribution of fragmentary transfer, as ORB+ gene sets that do not yield topologically discordant proteins or obligate edits have not been included.

## 4. Conclusion

Strains of *E. coli* and *Shigella* are more likely to exchange genetic material with their close relatives than with those more distantly related. As LGT is similarly biased at broader phyletic scale [6,7,13], together with other recent analyses [6–8,10,11], our results contribute to an emerging picture of relatedness bias across and at all taxonomic ranks within the prokaryotic domains. On the other hand, we find little evidence for bias favouring transfer among strains of *E. coli* and *Shigella* that share an environment or lifestyle.

In contrast, Smillie *et al*. [54] reported that among distantly related genomes, genetic exchange is structured by ecology more than by phylogeny, with preferential exchange among isolates that share ecologically similar environments. In general, phylogeny is expected to be important because genetic material exchanged among close relatives can be integrated via homologous recombination, and has greater compatibility with native host systems [41,55]. Moreover, shared evolutionary history is associated with mechanisms known to bias uptake of genetic material, including phage host infection biases, DNA uptake specificity and quorum sensing [56]. Our results suggest that within *E. coli*–*Shigella*, relatedness overrides shared ecology. LGT in this clade either transgresses environmental and lifestyle boundaries, or alters the organism-scale biology over time such that extant genomes cannot dependably be assigned to an exclusive lifestyle or ecotype. We favour the former explanation, as environmental and lifestyle annotations group coherently on the MRP supertree.

The edges we infer for obligate LGT form a densely connected graph, identifying the *E. coli–Shigella* clade as a GEC within which barriers to LGT are low. Bacterial lifestyle, habitat and phylogenetic relatedness do not pose substantial barriers to successful LGT, although transfer is biased to favour strains that are more closely related. More than one-third of donor–recipient pairs have exchanged fragments of genes, again emphasizing that whole genes are not privileged units of genetic transfer.

We have previously discussed the appropriateness of graph-based structures, including paths, cliques, near-cliques and transitively closed sets to define GECs, and expressed concern that GECs as cliques or near-cliques sets too high an evidentiary standard [5]. The DOLN (as based on obligate edits) reinforces this view.

## 5. Methods

We previously applied a computational workflow to reconstruct evolutionary histories and infer recombination breakpoints among 5282 putatively orthologous proteins in 27 *E. coli* and *Shigella* genomes [17]. Completely sequenced genomes were retrieved from NCBI, and whole-genome alignment was performed using the progressive Mauve algorithm of Mauve v. 2.3.0 [57,58]. The Mauve *export orthologs* function was then applied, yielding 5282 sets of positionally homologous protein-coding genes of size 4 ≤ *n* ≤ 27. Proteins sequences were aligned using ProbCons [59] and ambiguously aligned regions removed using GBLOCKS v. 0.91b [60].

Following alignment, 5282 phylogenetic trees were inferred using MrBayes v. 3.1.2 [37,61]. All their bipartitions with PP ≥ 0.95 were aggregated to generate an *E. coli*–*Shigella* reference tree, using MRP [38]. In parallel, a two-phase strategy [62] was implemented to detect recombination in the corresponding nucleotide alignments [17]. Classification of internal recombination breakpoints follows [52]. For further details, including parameter value settings for Mauve, ProbCons, GBLOCKS and MrBayes, see [17].

Topological discordance between the MRP supertree and individual query (protein) trees was assessed using EEEP [43] with a 95 per cent bootstrap collapse threshold and the strict reference treeratchet (-rR). Where an optimal solution could be found, EEEP reports the minimal set of subtree prune-and-regraft operations (*edits*) required to render the MRP supertree topologically consistent with a given query tree. The set of inferred *obligate* edits [42] was represented as a network in which nodes represent genomes (extant or inferred as ancestral from the MRP tree). An edge is drawn between genomes implicated as a donor–recipient pair by an obligate edit resolving incongruence for at least one protein set. Edges are labelled by the total number of incongruent protein sets that infer that obligate edit (LGT event). These analyses were implemented using custom Python scripts.

Supporting data (individual protein-family trees, inferred edits and help file) are available at http://bioinformatics.org.au/tools-data as ‘*E. coli–Shigella* 27 genomes LGT’.

## 6. Acknowledgements

We thank Aaron Darling for the *E. coli*–*Shigella* MAUVE alignment, and Cheong Xin Chan for breakpoint detection scripts. This work was supported by Australian Research Council grant CE0348221 and the University of Queensland. ES was supported by an Australian Postgraduate Award and a Queensland Government Smart State PhD Scholarship. Analyses were carried out at the National Computational Infrastructure National Facility.
